# Measurement of Plasma Galectin-3 Concentrations in Patients with Catheter Infections: A Post Hoc Retrospective Cohort Study

**DOI:** 10.3390/diagnostics12102418

**Published:** 2022-10-06

**Authors:** Simona Iftimie, Anna Hernández-Aguilera, Ana F. López-Azcona, Helena Castañé, Elisabet Rodríguez-Tomàs, Gerard Baiges-Gaya, Jordi Camps, Antoni Castro, Jorge Joven

**Affiliations:** 1 Department of Internal Medicine, Hospital Universitari de Sant Joan, Institut d’Investigació Sanitària Pere Virgili, Universitat Rovira i Virgili, 43204 Reus, Spain; 2 Unitat de Recerca Biomèdica, Hospital Universitari de Sant Joan, Institut d’Investigació Sanitària Pere Virgili, Universitat Rovira i Virgili, 43204 Reus, Spain

**Keywords:** C-reactive protein, central venous catheter, galectin-3, procalcitonin, urinary catheter

## Abstract

Catheter-related infections (CRIs) include catheter-associated urinary tract infections (CAUTIs) and central line-associated bloodstream infections (CLABSIs), and they are associated with high morbidity, mortality, and healthcare costs. The diagnosis of a CRI is made difficult by its non-specific symptoms. We aimed to investigate the factors influencing the plasma concentration of galectin-3 in catheter-bearing patients and to explore its potential usefulness as an index for CRIs. Circulating the concentrations of galectin-3, we measured the chemokine (C-C) motif ligand 2, procalcitonin, and C-reactive protein in 110 patients with a central catheter, in 165 patients with a urinary catheter, and in 72 control subjects. Catheter-bearing patients had higher concentrations (*p* < 0.001) of galectin-3 than the control group [central catheter: 19.1 (14.0–23.4) µg/L; urinary catheter: 17.1 (12.7–25.4) µg/L; control group: 6.1 (5.0–8.7) µg/L]. We identified chronic kidney disease as an independent determinant of galectin-3 concentrations in patients with a central catheter, and serum creatinine, cardiovascular disease, and number of days that the catheter was indwelling were identified as determinants in urinary catheter patients. We found that measuring galectin-3 concentrations in urinary catheter patients with a CRI was more accurate for diagnosis than the other parameters. We conclude that the measurement of galectin-3 concentration may be useful for assessing the inflammatory status of catheter-bearing patients and may contribute to the diagnosis of CRIs in those with a urinary catheter.

## 1. Introduction

The use of catheters is an integral part of modern healthcare, especially in aspects such as providing access to extracting samples for analysis, the administration of medication or parenteral nutrition, and the access for hemodialysis and hemodynamic monitoring. Two widely employed types of catheters in medicine are central venous catheters (CVCs) and urinary catheters (UCs). However, the use of these devices is not without complications; catheter-associated urinary tract infections (CAUTIs) and central line-associated bloodstream infections (CLABSIs) stand out, as they trigger a strong inflammatory reaction and are associated with increased morbidity, mortality, and healthcare costs [1,2]. Unfortunately, the clinical diagnosis of catheter-related infections (CRIs) are made difficult by the absence of specific signs and symptoms in many patients, including fever, chills, and hypotension [3]. Therefore, alterations in biochemical parameters related to infectious and inflammatory processes must be investigated with the aim of identifying biochemical markers that are capable of diagnosing infections in patients with indwelling urinary or central venous catheters. Several studies have proposed C-reactive protein (CRP), procalcitonin, or the chemokine (C-C motif) ligand 2 (CCL2) as markers of infection. However, their usefulness varies according to the clinical setting, and their application to patients with indwelling urinary or central venous catheters has not shown much utility, which is an unresolved issue [4,5].

Galectin-3 belongs to a family of β-galactoside binding proteins with a structure that allows them to participate in functions such as cellular and molecular recognition as well as adhesion [6]. Each galectin contains a C-terminal domain of 130 amino acids, termed a carbohydrate recognition domain, which is responsible for binding to galactose-containing sugar moieties. Galectin-3 also possesses a N-terminal domain with a unique short end continuing into a Pro-Gly-Ala-Tyr-rich repeat motif [7]. This protein has a molecular mass of 31 kDa, is strongly expressed by macrophages, and is a modulator of multiple biological functions, such as proliferation, macrophage chemotaxis, phagocytosis, neutrophil extravasation, neutrophil migration, apoptosis, vacuole lysis after infection, fibrogenesis, and angiogenesis [8,9,10]. The measurement of plasma galectin-3 concentration has been suggested as an index of inflammation and fibrosis in various diseases [11,12,13]. Several studies report that galectin-3 is a component of the innate immune system, participating in the organism’s defense mechanisms against infection, promoting macrophage and neutrophil survival, and promoting phagocytosis [14,15,16]. It is also able to bind to lipopolysaccharides from various types of bacteria, protecting hosts from endotoxin shock [17]. Galectin-3 is even a bactericide against Helicobacter pylori and is also a bacteriostatic against Streptococcus pneumoniae [9,18].

Therefore, the aim of this study was to investigate the factors influencing the plasma concentration of galectin-3 and to explore the potential usefulness of using this lectin as a biomarker for CRIs in hospitalized patients bearing a CVC or a UC.

## 2. Materials and Methods

### 2.1. Study Design

The study was carried out on patients hospitalized in the Department of Internal Medicine of the Hospital Universitari de Sant Joan in Reus, Spain, which is a 342-bed public care institution that provides medical care to an area of 250,000 inhabitants and is affiliated with Rovira i Virgili University. This was post hoc retrospective cohort study including new objectives derived from a previous prospective longitudinal study searching for biomarkers of catheter-related infections [19,20]. Patients were recruited between March 2011 and June 2013. We selected 110 patients with a CVC and 165 patients with a UC who were hospitalized in the Department of Internal Medicine or in the Intermediate Care Unit of our institution. The inclusion criterion was being an admitted patient with a catheter that had to be removed due to suspected CRI or because its use was no longer required. Exclusion criteria were being under 18 years of age, intermittent catheterization, or voluntarily withdrawing from the study. The CVCs were made of aliphatic polyurethane (Seldiflex trilumen deadult, Intersurgical España, Madrid, Spain; Lifecath PICC 5FR, Vygon SAU, Valencia, Spain). Urinary catheters were made of latex (Foley latex, Teleflex Medical SA, Madrid, Spain). At the time of catheter removal, a blood sample was obtained for the measurement of galectin-3, CRP, procalcitonin, and CCL2; urine samples and the catheter tips were also collected for microbiological analyses. The control group consisted of 72 healthy volunteers who participated in an epidemiological study conducted in our geographical area; the details of that study have been previously reported [21]. These samples belonged to a collection that was the result of an initiative of our research institute, which was conducted with the aim that a correctly performed control group was available for all our researchers. The participants were searched from the census and underwent a complete medical visit and blood test. These subjects had no clinical or analytical evidence of renal insufficiency, liver disease, infectious disease, neoplasia, or neurological disorders. The patients and controls were selected such that the different groups had the most similar possible age and sex distributions. Serum and plasma samples from all participants were stored in our Biobank at −80 °C until the time of the study. We recorded demographic data, bacteriological data, therapeutic data, comorbidities, and other acute or chronic infections. We also calculated the McCabe score as an index of clinical prognosis [22] and the Charlson index as a way of categorizing the patients’ comorbidities [23]. Of the participants, 116 (42%) were hospitalized for surgery, 72 (26%) were hospitalized for an infectious disease, and the remaining 87 (32%) had various other clinical conditions. In patients with a CVC, the location of the catheter tip was brachial (peripherally inserted central catheters, PICC) in 47 patients (43%), subclavian in 51 (46%), jugular in 9 (8%), and femoral in 3 (3%).

Patients with an acute concomitant infection (ACI) suffered from an infection (abdominal abscess, pneumonia, etc.) that was not related to an infected catheter. Of these patients, 61 (22%) had an ACI without a CRI, 29 (11%) had a CRI without an ACI, and 24 (9%) had both infections simultaneously. The study was conducted in accordance with the Declaration of Helsinki and was approved by the Ethics Committee (Institutional Review Board) of the Hospital Universitari de Sant Joan (12proj2. 10/12/23. Approved 29 December 2010). All subjects provided written informed consent to participate in the study on the understanding that anonymity of data was guaranteed.

The desired primary outcome was to know if the determination of galectin-3 concentrations could be useful for the diagnosis of CRIs. The secondary outcomes were: (1) to investigate the differences between the circulating concentrations of galectin-3 in patients with catheters compared with the control subjects; (2) to investigate the relationships between galectin-3 levels and the pathological background of patients; (3) to study the relationships between galectin-3 and the indices of inflammation CCL2, CRP, and procalcitonin.

### 2.2. Biochemical and Microbiological Analyses

Blood samples were obtained at the time of catheter removal due to suspected catheter-related infection. The ethylene diamine tetraacetate (EDTA) plasma concentrations of galectin-3 and CCL2 and the serum concentrations of procalcitonin were determined by an enzyme-linked immunosorbent assay (R&D Systems^®^, Minneapolis, MN, USA; Biovendor, Brno, Czech Republic; and Peprotech, London, UK, respectively). Serum CRP concentrations were measured by a high-sensitive solid phase chemiluminescent immunoassay (Immulite^®^ 2000 High Sensitivity CRP, Siemens, Munich, Germany) in an Immulite^®^ 2000 Xpi automated analyzer (Siemens). Serum creatinine concentrations were analyzed using the Jaffe method in a Cobas^®^ 8000 modular platform (Roche Diagnostics, Basel, Switzerland).

Catheters were cultured using standard methods and classified into four categories: uninfected, when there was no evidence of infection and cultures were negative; catheter colonization, when we observed a growth of a microorganism higher than 15,000 colony-forming units (CFU)/L from the catheter tip with no clinical evidence of infection; insertion site infection, when there was erythema or induration within 2 cm of the catheter exit site, in the absence of a concomitant bloodstream infection; and catheter-related bacteremia, when the blood cultures and catheter tip culture were positive for the same microorganism. Identification and susceptibility tests of the isolated strain were performed by an automated microdilution (MicroScan WalkAway, Siemens Healthcare, Erlangen, Germany) and/or disk diffusion method, which were conducted with complementary biochemical tests depending on the type of microorganism.

The culture of the urine samples was carried out in accordance with the recommendations of the Spanish Society of Infectious Diseases and Clinical Microbiology. One µL of homogenized urine was seeded on the surface of a plate with a CLED culture medium, and another on a blood agar medium (AS), making a streak in the center. Next, the inoculum was spread through perpendicular streaks. Once the culture media had been inoculated, they were incubated at 35–37 °C in aerobiosis for 24–48 h. After incubating the plates, we counted the CFUs. In general, a positive culture was considered when the count was ≥10^8^ CFU/L of any microorganism in a pure culture. Cultures with lower counts were interpreted as positive depending on the type of microorganism isolated, presence of pyuria in the sediment, and analytical and clinical data according to the criteria of the microbiologist. Subsequently, the identification and antibiogram of the isolated microorganism(s) was performed using the MicroScan WalkAway automated microdilution system and/or disk diffusion, which were conducted with complementary biochemical tests depending on the type of microorganism.

### 2.3. Statistical Analyses

All calculations were made using the SPSS 24.0 statistical package (SPSS Inc., Chicago, IL, USA). Because most of the studied variables had non-Gaussian distributions, differences between any two groups were assessed using the Mann–Whitney U test. Qualitative data were analyzed using the χ^2^ test, and correlations were analyzed using Spearman’s ρ test. The combined effect of the clinical and demographic characteristics on plasma galectin-3 concentrations was assessed by a multiple regression analysis. The diagnostic accuracy of the measured biochemical variables was assessed using the receiver operating characteristics (ROC) curves [24]. Results are shown as medians and interquartile ranges (IQR).

## 3. Results

Patients with a UC were significantly older, and the percentage of males was higher than in patients with a CVC. Patients with a UC tended to more often have arterial hypertension, cardiovascular disease, chronic kidney disease, and/or treatment with antibiotics, and had neoplasia and/or treatments with immunosuppressive drugs significantly less often. The McCabe score indicated a better prognosis in UC patients, and the Charlson index was similar between groups. We observed that 13 patients with a central catheter and 16 with a urinary catheter had temperatures greater than 37 °C; none of them had hematuria. They did not show any significant difference in the incidence of CRI or acute concomitant infection (ACI) (Table 1).

Patients with a CVC or UC had significantly higher concentrations of galectin-3, CRP, procalcitonin, and CCl2 than the control group (Table 2). 

**Figure 1:**
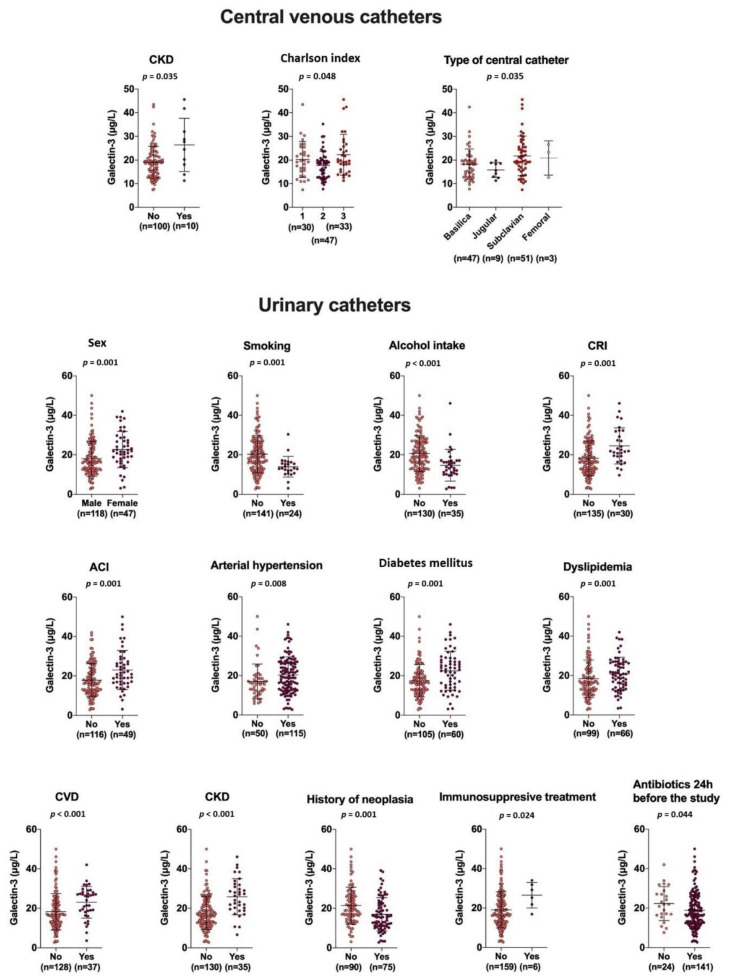
Influence of demographic and clinical variables on plasma galectin-3 concentrations in catheter-bearing patients. Statistical analyses used the χ^2^ test or the Kruskal–Wallis test. CKD: chronic kidney disease; CRI: catheter-related infection; CVD: cardiovascular disease.

Bivariate statistical analysis showed patients with a CVC had higher plasma galectin-3 concentrations in those with chronic kidney disease, a higher Charlson index, or that the catheter was in the subclavian vein (this last observation may be related to the fact that subclavian catheters are more susceptible to infection than peripheral catheters, as they are shorter and closer to the otorhinopharyngeal system). In contrast, patients with UC had higher galectin-3 concentrations in women and in patients with a CRI, ACI, arterial hypertension, diabetes mellitus, dyslipidemia, cardiovascular or chronic kidney disease, neoplasia, immunosuppressive treatment, or had received antibiotics; lower galectin-3 concentrations were found in smokers or people who usually drink alcohol (Figure 1).

**Figure 2:**
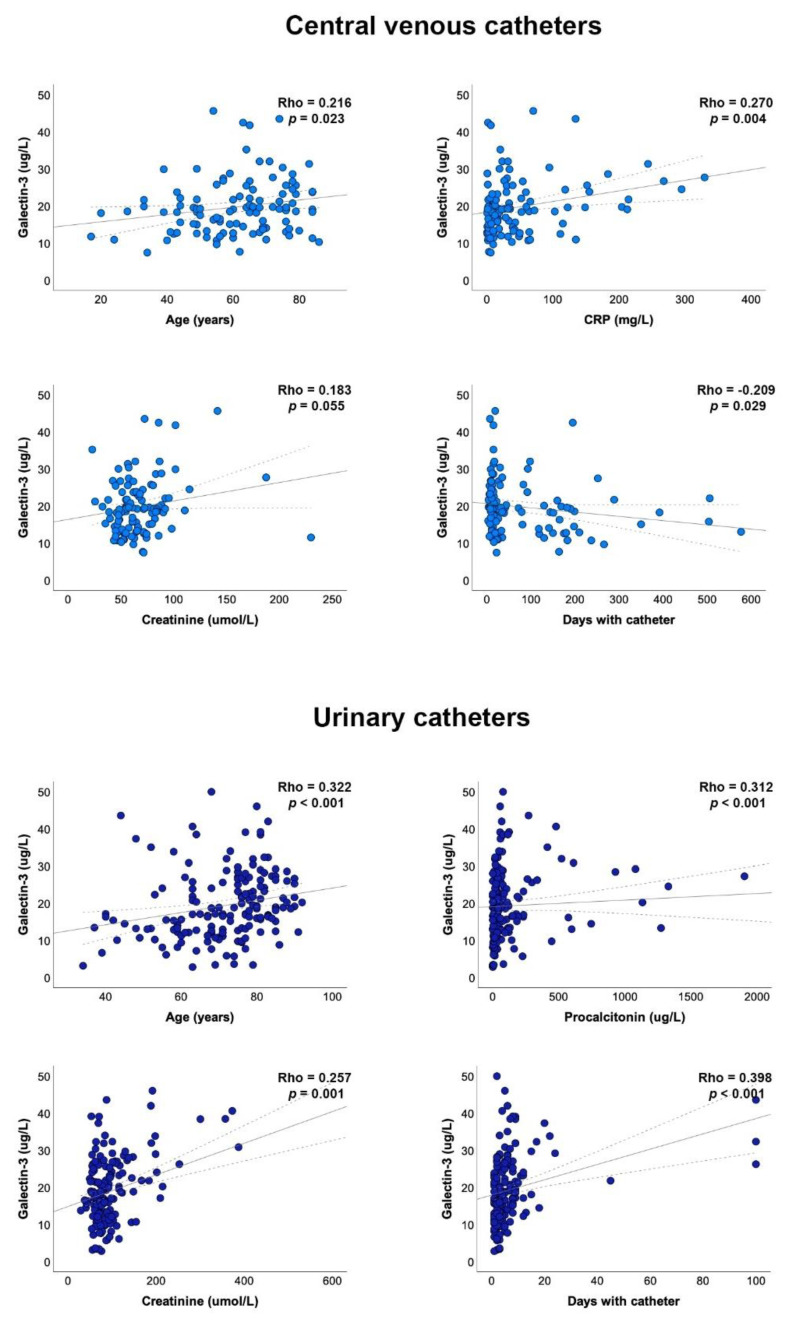
Relationships between the selected clinical and biochemical variables and plasma galectin-3 concentrations in the catheter-bearing patients. Statistical analyses used Spearman’s Rho test.

In patients with a CVC, significant direct correlations were observed with age and the CRP and creatinine concentrations, and an inverse correlation with the number of days that patient carried the catheter. In UC patients, significant direct correlations were observed with age, procalcitonin, creatinine concentrations, and the number of days that patient carried the catheter (Figure 2). 

**Figure 3:**
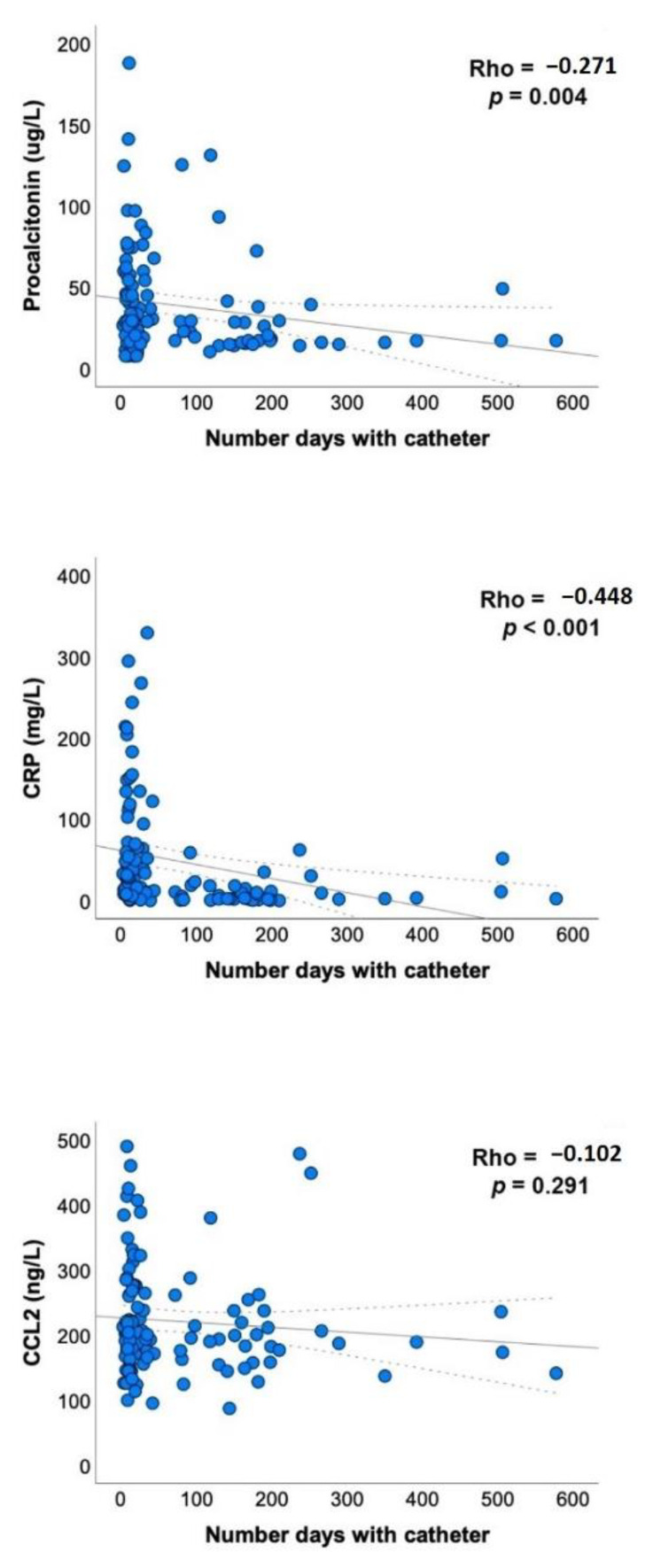
Relationships between the number of days the catheter was indwelling and the circulating levels of procalcitonin, C-reactive protein (CRP)m and the chemokine (C-C) motif ligand 2 (CCL2) in patients with a central venous catheter. Statistical analyses used Spearman’s Rho test.

A surprising finding of the present study was that there was the negative correlation between galectin-3 concentrations and the number of days that the catheter was indwelling in CVC patients. To understand this association, we verified that there were also negative correlations between the number of days and the circulating concentrations of CRP and procalcitonin (Figure 3), which indicates a decrease in the degree of inflammation.

Among the 23 patients with a CVC and CRI, 11 (47.8%) had catheter-only colonization, 9 (39.1%) had infection at the insertion site, and 3 (13.0%) had catheter-related bacteremia. Among UC patients, the urine culture was negative in 135 patients (82%), positive for gram-negative bacteria in 13 patients (7.8%), positive for gram-positive bacteria in 8 patients (4.8%), and positive for assorted fungi in 9 patients (5.4%). The infection rate in our patients was relatively low because we had a high number of patients in the postoperative period of prostate resection and thus had the catheter for a few days, meaning the risk of infection was low. The identified strains and colony forming units (CFU)/L are shown in Table 3. We did not observe any significant difference in the circulating levels of galectin-3 depending on the identified microorganism.

The number of factors associated with galectin-3 concentration in our patients was high, but as all these factors may also present associations between them, we performed a multiple regression analysis to identify statistically independent determinants. We found that the presence of chronic kidney disease was an independent determinant of plasma galectin-3 concentrations in CVC patients (Table 4) and that serum creatinine concentration, cardiovascular disease, and the number of days the catheter was indwelling served as independent determinants of plasma galectin-3 concentrations in UC patients (Table 5).

**Figure 4:**
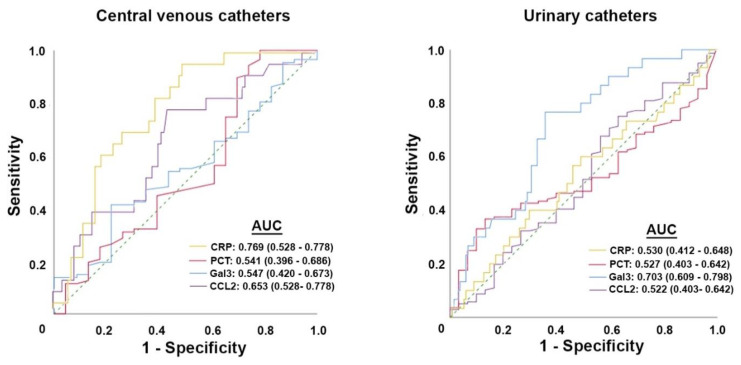
Receiver operating characteristics (ROC) plot of selected biochemical variables in the catheter-bearing patients with respect to the presentation of catheter-related infections. AUC: area under the curve; CRP: C-reactive protein; Gal3: galectin-3; PCT: procalcitonin.

Finally, we wanted to explore the possibility that galectin-3 determination was a reliable index for the diagnosis of CRIs, and we wanted to compare it with the effectiveness of CRP, prolactin, and CCL2 determinations. When comparing the diagnostic accuracy of the ROC curves, we found that the order of effectiveness was CRP > CCL2 > procalcitonin > galectin-3 in patients with a CVC, and galectin-3 > CRP ≈ procalcitonin ≈ CCL2 in patients with UC (>: greater than; ≈: similar). The area under the curve of galectin-3 was far greater than that of the other parameters in UC patients (Figure 4). In UC patients, at galectin-3 = 15 ng/mL, sensitivity was 90% and specificity was 41%. Values below 15 ng/mL were very unlikely to indicate CRIs. In our series, 56 out of 165 patients (33.9%) in theory would not necessarily need confirmation by urine culture.

## 4. Discussion

Our results show that catheter-bearing patients have higher plasma galectin-3 concentrations than what was observed in the healthy population. The increase in the levels of this protein is very similar to that of the concentrations of CRP, procalcitonin, and CCL2, and they present significant correlations, which suggests that the increase in the concentrations of galectin-3 is related to the inflammatory processes subsequent to the diseases of these patients. There are many diseases related to the use of catheters; therefore, there are multiple factors that can influence the increase in galectin-3. Indeed, the mere use of catheters can produce an inflammatory reaction. Catheters are made from a variety of materials combined with different chemicals, and it seems that these chemical substances can dissolve from the catheter material, causing an inflammatory reaction [25,26]. However, the factors that influence galectin-3 concentrations in our study are different in patients with a CVC or UC. In the former, high levels of galectin-3 are associated with the presence of chronic kidney disease, higher age, and higher serum CRP concentrations. Patients with higher Charlson index values show slightly higher galectin-3 values, indicating a possible effect of associated comorbidities. Likewise, the levels of this protein are also somewhat higher in patients in whom the catheter was placed in the subclavian vein. On the other hand, in patients with a UC, galectin-3 concentrations were associated with multiple factors, including demographic variables, several associated diseases, treatments, and the circulating concentrations of procalcitonin and creatinine. If we search for factors common to all types of catheters, we can define age, the level of inflammation (identified by correlations with CRP or procalcitonin), and renal function (identified by the presence of chronic kidney disease or serum creatinine levels) as common to both CVC and UC patients. Several studies have shown that the plasma concentration of galectin-3 is strongly dependent on renal function [27,28,29,30]. Other studies have shown that the levels of this protein correlate with the glomerular filtration rate in patients with a cirrhosis of the liver [31] or polycystic ovary [32]. High concentrations of galectin-3 in plasma have been associated with a risk of chronic kidney disease and are reduced using hemodialysis treatments [33,34,35]. It has been reported that an increase in the circulating concentration of galectin-3 (as can be observed in some inflammatory diseases) precedes the decrease in glomerular filtration. Likewise, the development of chronic kidney disease produces a decrease in the clearance of galectin-3, leading to a further increase in the circulatory system [36,37].

Taken together, our data and the reports by other authors indicate that renal function is an important determinant of the circulating levels of galectin-3. We found a significant positive association between galectin-3 concentrations and the number of days that the catheter was indwelling in patients with a UC. This finding may have considerable clinical relevance, as the long-term localization of catheters in situ can result in the formation of bacterial biofilms that become resistant to antibiotic treatments [38]. Catheter insertion usually provokes inflammation, which can in turn result in a hampered antimicrobial capacity in the host’s immunity due to the effort of immune cells being directed at degrading the foreign material. Ineffective clearance by immune cells is a perfect opportunity for bacteria to attach and form a biofilm [39]. The longer the catheter is in place, the more opportunities the bacteria have to form a biofilm and increase the inflammatory reaction [40]. Biofilms can develop because of the physicochemical interactions between microorganisms and the surface of the supporting catheter, along with specific greater adhesions found on the cell surface or filamentous structures of the bacterial wall, such as the pili and fimbriae. Once attached, bacteria synthesize an extracellular polymeric matrix that provides a structural support for the colony. The outcome is antimicrobial resistance, either because of the physical barrier to the entry of therapeutic drugs, or because of the difficulty that the antibacterial agents encounter when diffusing into the biofilm [41].

Our results suggest that the measurement of galectin-3 levels can help diagnose CRI in patients with an indwelling urinary catheter better than that of CRP and procalcitonin levels. Currently, the diagnosis of bacterial infections in these patients is hampered by the lack of adequate biomarkers. To confirm or rule out a suspected urinary tract infection, blood, tests, urine tests, and sometimes blood cultures and radiographs should be routinely performed. Urine culturing is the gold standard method but requires a certain amount of time [42], and sometimes clinicians must look for alternatives to quickly diagnose and treat patients. Although simpler and more rapid, the urinary dipstick analysis has a lack of sensitivity [43]. Hence, the quest for the identification of appropriate biomarkers for a rapid and correct diagnosis of CRIs has been an unresolved issue. The most common biochemical markers of infection/inflammation are the CRP and procalcitonin levels. However, these parameters are nonspecific, as their values may increase in response to any type of inflammatory stimulus, such as autoimmune diseases, neoplasms, or stress situations. Indeed, in our study, although urinary catheter patients had higher concentrations of CRP and procalcitonin than the control subjects, the ROC curves showed that these parameters were unable to distinguish between patients with a CRI and those without a CRI. The measurement of galectin-3 levels has a higher diagnostic accuracy and could add improvement to the lack of biochemical markers. A combined sensitivity of 90% with a specificity of 41% would result in a saving of about a third of the urine bacterial cultures from catheterized patients, leaving two-thirds of the diagnoses to result from culturing. Therefore, the usefulness of using galectin-3 as a marker of CRIs is limited, and its exclusive determination cannot be proposed as a diagnostic biomarker for CRIs. However, its addition to the battery of analytical tests can be of clinical utility.

We are aware that our study had several limitations. The patient groups were heterogeneous. The medical reasons that determine the catheterization of a patient are diverse, and there are multiple underlying pathologies. There are also a variety of microbial agents that can infect catheters. Furthermore, our study design implies that there are some grounds for possible bias. First, some of the patients that were studied had no symptoms or signs of catheter infection. However, this potential bias is not in favor of our hypothesis, but rather goes against it as these patients would have lower galectin-3 levels. In addition, the fact that the patients did not present clinical signs of infection does not necessarily mean that they did not have an infection; therefore, their exclusion would possibly induce another type of bias, this time one that would favor our hypothesis. Second, we included patients with ACIs who may have higher levels of galectin-3. We think that despite this circumstance, including these patients is interesting, as one of the causes of catheterization in patients is concomitant infectious diseases. Our results indicate that having an ACI does not influence galectin-3 levels in patients with a CVC, but it does in those with a UC (Figure 1). We believe that this diversity reflects the usual clinical practice faced by physicians in a general hospital, and the metabolic reactions, oxidative stress, and inflammation observed are similar regardless of the cause. Finally, the retrospective design of our study has the limitation that there was no prior randomization of the sample, which prevents us from guaranteeing that the groups being compared are comparable. Furthermore, this limitation also suggests that although associations can be observed, cause–effect relationships cannot be established. Future prospective multicenter studies should be carried out with a much larger number of patients to fully discern the role played by galectin-3 levels in CRIs and its possible utility as a biomarker.

## 5. Conclusions

We found that age, degree of inflammation, and renal function are the main determinants of increased plasma galectin-3 concentrations in catheter-bearing patients. We also suggest that the determination of this parameter may contribute to the diagnosis of CRIs in patients with a UC.

## Figures and Tables

**Table 1:** Demographic and clinical characteristics of the patients and control group.

Feature		Control Group (*n* = 72)	CVC (*n* = 110)	UC (*n* = 165)
Age, years		63 (61–69)	63 (50–74)	75 (63–81) ^c,f^
Sex (male), *n* (%)		47 (65.3)	54 (49.1) ^a^	118 (71.5) ^f^
Smoking, *n* (%)		26 (36.1)	25 (22.7) ^b^	24 (14.5) ^c^
Alcohol intake, *n* (%)		36 (50.0)	15 (13.6) ^c^	35 (21.2) ^c^
Catheter-related infection, *n* (%)		NA	23 (20.9)	30 (18.2) ^d^
Acute concomitant infection, *n* (%)		NA	36 (32.7)	49 (29.7)
Hypertension, *n* (%)		5 (6.9)	57 (51.8) ^c^	115 (69.7) ^c,e^
Diabetes mellitus, *n* (%)		0	32 (29.1)	60 (36.4)
Dyslipidemia, *n* (%)		0	35 (31.8)	66 (40.0)
Cardiovascular disease, *n* (%)		0	11 (10.0)	37 (22.4) ^e^
Chronic kidney disease, *n* (%)		0	10 (9.1)	35 (21.2) ^e^
Neurological vascular disease, *n* (%)		0	12 (10.9)	12 (7.3)
COPD, *n* (%)		0	11 (10.0)	31 (18.8) ^a^
History of neoplasia, *n* (%)		0	71 (64.5)	75 (45.5) ^e^
Immunosuppressive treatment, *n* (%)		0	45 (40.9)	6 (3.6) ^f^
Antibiotics 24 h before the study, *n* (%)		0	51 (46.4)	141 (85.5) ^f^
Charlson index	No comorbidity, *n* (%)	NA	30 (27.3)	67 (40.6)
	Low comorbidity, *n* (%)	NA	47 (42.7)	54 (32.7)
	High comorbidity, *n* (%)	NA	33 (30.0)	44 (26.7)
McCabe index	RFD, *n* (%)	NA	9 (8.2)	18 (10.9)
	UFD, *n* (%)	NA	28 (25.5)	13 (7.9) ^f^
	NFD, *n* (%)	NA	73 (66.4)	134 (81.2) ^f^
Days with catheter		NA	22 (10–130)	4 (2–7) ^f^

COPD: chronic obstructive pulmonary disease; CVC: central venous catheter; NA: not applicable; NFD: nonfatal disease; RFD: rapidly fatal disease; UC: urinary catheter; UFD: ultimately fatal disease. Data are shown as medians and interquartile ranges or number of cases and percentages. ^a^: *p* < 0.05, ^b^: *p* < 0.01, ^c^: *p* < 0.001, with respect to the control group; ^d^: *p* < 0.05, ^e^: *p* < 0.01, ^f^: *p* < 0.001, with respect to the CVC patients. Statistical analyses were performed using the Mann–Whitney U test (quantitative) or Χ^2^ test (qualitative).

**Table 2:** Selected biochemical variables in the catheter-bearing patients and control group. Statistical analyses used the Mann–Whitney U test. Results are shown as the medians and IQRs.

Variable	Control Group (*n* = 72)	CVC (*n* = 110)	UC (*n* = 165)
Galectin-3 (µg/L)	6.1 (5.0–8.7)	19.1 (14.0–23.4) ^a^	17.1 (12.7–25.4) ^a^
CCL2 (ng/L)	152.3 (130.3–175.3)	201.1 (165.2–261.8) ^a^	188.9 (152.7–250.2) ^a^
Procalcitonin (µg/L)	<DLA	29.1 (17.3–46.2) ^a^	41.4 (16.1–104.4) ^a,b^
C-reactive protein (mg/L)	1.2 (0.4–2.4)	22.1 (6.4–59.3) ^a^	24.6 (13.4–47.8) ^a^

CCL2: chemokine (C-C motif) ligand 2; CVC: central venous catheter; DLA: detection limit of the assay; UC: urinary catheter. ^a^ *p* < 0.001 with respect to the control group; ^b^ *p* < 0.05 with respect to the CVC group. Procalcitonin values in the control group have been considered as zero for statistical purposes.

**Table 3:** Microorganisms isolated in UC patients with catheter-related infections.

PIC	Microorganism	CFU/L
8	*Enterococcus faecalis*	10^8^
10	*Staphylococcus epidermidis*	>10^8^
11	*Staphylococcus epidermidis, Corynebacterium*	>10^8^
15	*Enterococcus faecalis*	5 × 10^7^–10^8^
17	*Escherichia coli*	>10^8^
24	*Pseudomonas aeruginosa*	>10^8^
27	*Pseudomonas aeruginosa*	5 × 10^7^–10^8^
33	*Raoultella planticola, Escherichia coli*	>10^8^
53	*Candida parapsilosis*	5 × 10^7^–10^8^
54	*Candida albicans*	>10^6^
63	*Providencia stuartii*	>10^8^
65	*Escherichia coli*	5 × 10^7^–10^8^
66	*Enterococcus faecalis*	5 × 10^7^–10^8^
83	*Candida albicans*	10^8^
93	*Escherichia coli*	>10^8^
96	*Enterococcus faecalis*	3 × 10^7^–4 × 10^7^
110	*Candida albicans*	4 × 10^7^–5 × 10^7^
133	*Escherichia coli*	5 × 10^7^–10^8^
135	*Escherichia coli*	>10^8^
137	*Enterococcus faecalis*	3 × 10^7^–4 × 10^7^
138	*Candida albicans*	>10^6^
142	*Pseudomonas aeruginosa*	5 × 10^7^–10^8^
144	*Enterococcus faecalis*	4 × 10^7^–5 × 10^7^
146	*Candida albicans*	>10^6^
147	*Candida albicans*	>10^8^
155	*Pseudomonas aeruginosa*	>10^8^
167	*Escherichia coli*	>10^8^
168	*Enterococcus faecalis*	5 × 10^7^–10^8^
170	*Candida albicans*	4 × 10^7^–5 × 10^7^
173	*Candida albicans*	>10^8^

**Table 4:** Multiple regression analysis of the combined influence of clinical characteristics and biochemical variables on plasma galectin-3 concentrations in patients with a central venous catheter. Model summary: r = 0.400; *p =* 0.005.

Variable	B	SE	β	*p*-Value
Constant	12.865	3.662	-	0.001
Age	0.050	0.049	0.100	0.314
C-reactive protein	0.017	0.011	0.155	0.119
Chronic kidney disease	6.098	2.647	0.236	0.023
Charlson index	0.225	1.064	0.023	0.833
Days with catheter	−0.001	0.008	−0.008	0.948
Type of catheter	0.993	0.910	0.133	0.278

Coefficient B indicates the number of units that the dependent variable increases by for each increase in the unit of the independent variable. The coefficient β is the standardized coefficient B. SE is the standard error.

**Table 5:** Multiple regression analysis of the combined influence of clinical characteristics and biochemical variables on plasma galectin-3 concentrations in patients with an indwelling urinary catheter. Model summary: r = 0.674; *p* < 0.001.

Variable	B	SE	β	*p*-Value
Constant	10.043	4.373	-	0.022
Age	0.089	0.053	0.126	0.098
Sex	−2.851	1.450	−0.146	0.051
Smoking	−1.134	1.784	−0.045	0.626
Alcohol intake	−1.633	1.491	−0.076	0.275
Chemokine (C-C motif) ligand 2	−0.009	0.006	−0.135	0.110
Procalcitonin	0.001	0.002	0.045	0.512
Creatinine	0.045	0.009	0.413	<0.001
Catheter-related infection	2.398	1.617	0.105	0.140
Acute concomitant infection	1.980	1.429	0.102	0.168
Hypertension	0.359	1.382	0.019	0.795
Diabetes mellitus	2.219	1.310	0.121	0.090
Dyslipidemia	−1.138	1.256	−0.063	0.366
Cardiovascular disease	2.894	1.452	0.137	0.048
History of neoplasia	−0.675	1.401	−0.038	0.631
Immunosuppressive treatment	1.877	3.043	0.040	0.538
Antibiotics 24 h before the study	−0.484	1.761	−0.019	0.784
Days with catheter	0.131	0.042	0.203	0.002

The coefficient B indicates the number of units that the dependent variable increases by for each increase in the unit of the independent variable. The coefficient β is the standardized coefficient B. SE is the standard error.

## Data Availability

Data are available from the corresponding author upon request.
